# p53-Dependent and Cell Specific Epigenetic Regulation of the *Polo-like kinases* under Oxidative Stress

**DOI:** 10.1371/journal.pone.0087918

**Published:** 2014-01-31

**Authors:** Alejandra Ward, John W. Hudson

**Affiliations:** Department of Biology, University of Windsor, Windsor, Ontario, Canada; Weizmann Institute of Science, Israel

## Abstract

The polo-like kinase (PLKs) family, consisting of five known members, are key regulators of important cell cycle processes, which include mitotic entry, centrosome duplication, spindle assembly, and cytokinesis. The *PLKs* have been implicated in a variety of cancers, such as hepatocellular carcinoma (HCC), with PLK1 typically overexpressed and PLKs 2–5 often downregulated. Altered expression of the PLKs in malignancy is often correlated with aberrant promoter methylation. Epigenetic marks are dynamic and can be modified in response to external environmental stimuli. The aim of our study was to determine if oxidative stress, a common feature of solid tumours, would induce changes to the promoter methylation of the *PLKs* resulting in changes in expression. We examined the promoter methylation status *via* MSP and subsequent expression levels of the *PLK* family members under exposure to hypoxic conditions or reactive oxygen species (ROS). Interestingly, murine embryonic fibroblasts exposed to hypoxia and ROS displayed significant hypermethylation of *Plk1* and *Plk4* promoter regions post treatment. Corresponding proteins were also depleted by 40% after treatment. We also examined the HCC-derived cell lines HepG2 and Hep3B and found that for *PLK1* and *PLK4*, the increase in hypermethylation was correlated with the presence of functional p53. In p53 wild-type cells, HepG2, both *PLK1* and *PLK4* were repressed with treatment, while in the p53 null cell line, Hep3B, PLK4 protein was elevated in the presence of hypoxia and ROS. This was also the case for ROS-treated, p53 null, osteosarcoma cells, Saos-2, where the *PLK4* promoter became hypomethylated and protein levels were elevated. Our data supports a model in which the *PLKs* are susceptible to epigenetic changes induced by microenvironmental cues and these modifications may be p53-dependent. This has important implications in HCC and other cancers, where epigenetic alterations of the *PLKs* could contribute to tumourigenesis and disease progression.

## Introduction

The polo-like kinases (*PLKs*) have been implicated in a variety of solid and hematopoietic tumours, which include B-cell lymphoma, hepatocellular carcinoma (HCC), head and neck squamous carcinoma, colorectal cancers, and most recently gallbladder cancer, just to name a few [1]–[5]. Moreover, their deregulation is often associated clinically with poor prognosis, such as the case of PLK1 overexpression in non-small cell lung carcinoma and head and neck squamous carcinoma, or downregulation of Plk4 in HCC [3], [6], [7]. Recently, we and others, have determined that the polo-like kinases, which are cell cycle regulated serine/threonine kinases, are susceptible to aberrant DNA methylation in many of the tumour types described above [1], [8]–[10]. Aberrant promoter methylation of *PLK1-4* have been implicated in hepatocellular carcinoma [9], [10], while *PLK2* promoter hypermethylation has been detected in hematologic malignancies such as acute myeloid leukemia and B-cell lymphoma, as well as in ovarian cancers [1], [8], [11]. Interestingly, the recently discovered *PLK5*, has tumour suppressor properties, and it is often hypermethylated in glioblastoma [12]. Given that these kinases, which are highly conserved among species, play crucial roles in important cell cycle events such as spindle pole assembly, the DNA damage response, G2/M transitions, and cytokinesis [6], [13], [14], proper regulation of these proteins is essential for the maintenance of genomic integrity and the prevention of genomic instability. Therefore, the underlying question is what is prompting the aberrant epigenetic regulation of the polo-like kinases in a variety of cancer types?

It has been established that the microenvironment plays a significant role in the initiation and progression of tumourigenesis. The cellular microenvironment provides a platform from which bidirectional molecular cues can be exchanged. This topographical information can direct cellular phenomena which include growth, cellular differentiation, and division. The aberrant alterations in the microenvironment can confer tumourigenicity through direct genetic mutations, but more so *via* epigenetic plasticity [15], [16]. Oxidative stress, in the form of reactive oxygen species (ROS) and hypoxia, are components of the tumour microenvironment, and have been shown to be causative agents of abnormal, epigenetically-induced gene expressions in a variety of tumour types [17]–[19]. Studies have also revealed that several tumour suppressors and cell cycle regulators such as *p14ARF*, *p16INK4a*, and *BRCA1* are susceptible to epigenetic silencing through DNA hypermethylation or histone modification in the presence of oxidative stress [19], [20]. The purpose of this study was to examine the susceptibility of individual *PLK* regulation through epigenetic modifications in response to oxidative stress in the form of either ROS or hypoxia. Here we have determined that the polo-like kinases are indeed epigenetically modified in the presence of oxidative stress, though in a cell type-dependent and p53-dependent manner. Furthermore, we have determined that *Plk4* heterozygosity may play a role in the epigenetic regulation of *Plk1* in response to oxidative stress.

## Results and Discussion

### *Plks* are subject to epigenetic modification under hypoxic conditions in normal and tumour-derived cells *in vitro*

Hypoxia has been established as a characteristic of the solid tumour microenvironment and has been shown to promote cell migration and cell transformation [21], [22]. The primary mediator of the cellular response to hypoxia is hypoxia inducible factor 1α (Hif1α) which is responsible for the transcriptional regulation of several key genes, such as vascular endothelial growth factor (VEGF) [23] and metabolic components such as nitric oxide (NO) which are important for the cellular adaptation to a hypoxic environment [24]. More recently, Hif1α has been shown to indirectly modify epigenetic marks on histone tails leading to varying levels of transcriptional activation and repression through histone deactylatase (HDAC) recruitment and modification of the H3K9 methylation marks [25].

**Figure 1:**
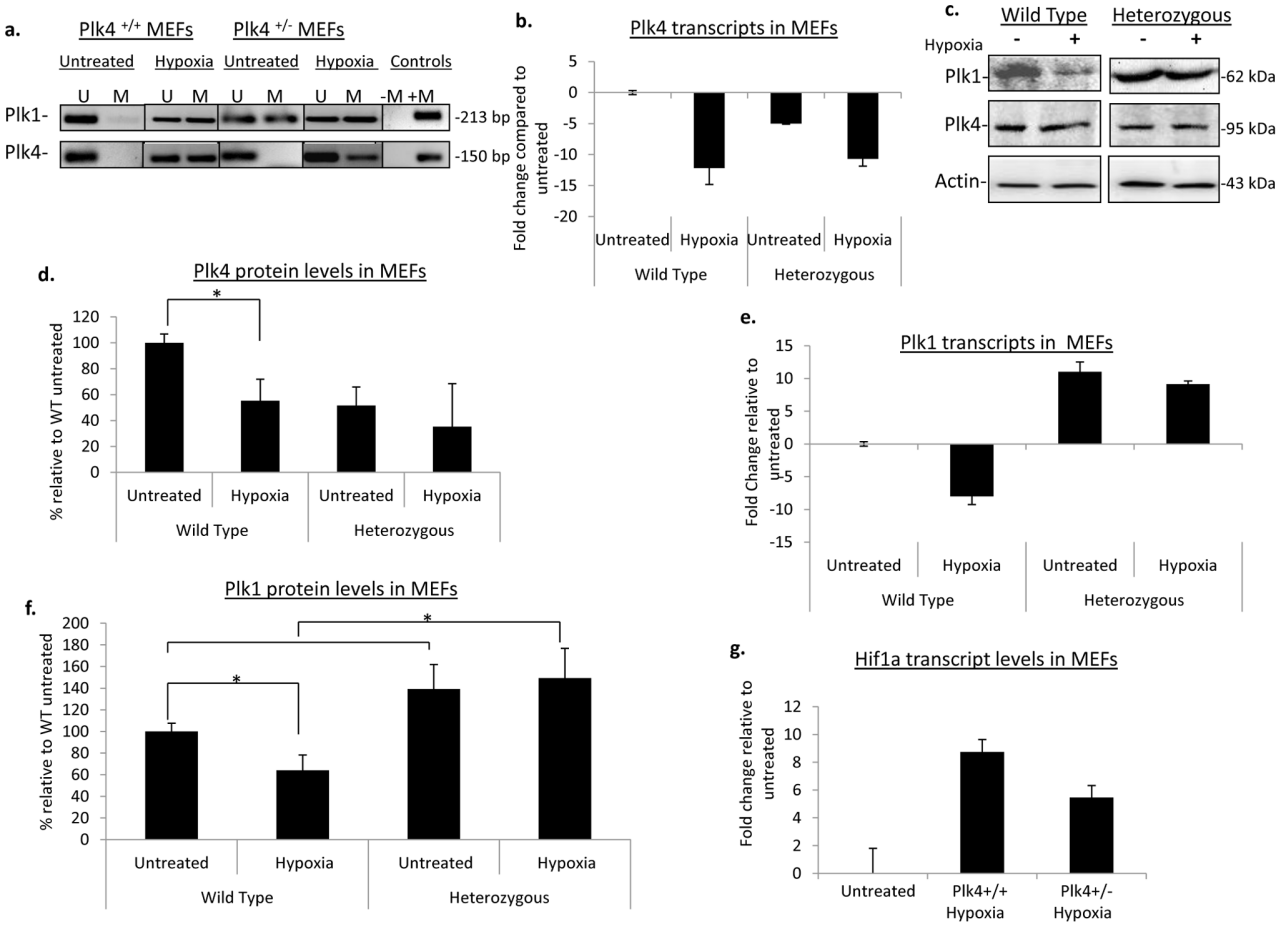
Aberrant methylation of *plk1* and *plk4* promoter regions in MEFs under hypoxic stress. (a) DNA extracted from mouse embryonic fibroblasts grown under hypoxic conditions was bisulfite treated and then assessed for promoter methylation of *Plk1* and *Plk4* using methylation specific PCR; U = unmethylated, M = methylated. Fully methylated NIH 3T3 DNA was used as a positive control (+M), no template was added to the negative control (−M). (b) Plk4 transcripts were assessed using qPCR. Transcript levels were normalized to the wild type untreated sample. All qPCR data is representative of the mean value of three independent experiments and error bars represent +/− SD. (c) Western blot analysis to examine protein levels of Plk1 and Plk4 post hypoxic treatment. (−) represents the lysates from untreated cells, (+) lysates from cells were grown in the presence of hypoxia. (d) Densitometric analysis normalized to the levels of the wild-type untreated cells. Error bars represent +/− SD from three independent experiments. (e) The fold change of plk1 transcripts normalized to the respective untreated transcripts. (f) The percent of Plk1 protein expression relative to the untreated wild-type cells. * denotes significance with p<0.05. (g) RNA extracted from MEFs along with real-time PCR was used to determine Hif1α transcripts post hypoxia treatment.

We have previously shown that *Plk4* heterozygosity increases the susceptibility of *Plk4* promoter methylation in an *in vivo* murine HCC model [10], therefore we wanted to determine whether *Plk4* heterozygosity impacted *Plk* promoter methylation under oxidative stress. First, wild type (*Plk4^+/+^*) and heterozygous (*Plk4^+/−^*) murine embryonic fibroblasts (MEFs) were cultured in a hypoxia chamber flooded with 2% oxygen and incubated for 18 hours in order to determine whether the exposure of cells to hypoxia results in the modification of *Plk* gene expression through epigenetic means. After the treatment, methylation specific PCR (MSP) was performed in order to examine the methylation status of the *Plks*. We did observed *Plk4* promoter methylation upon hypoxia treatment, regardless of genotype (Fig. 1a). Furthermore, corresponding Plk4 transcripts were decreased by approximately 12-fold compared to the untreated in both *Plk4^+/+^* and *Plk4^+/−^* MEFs under hypoxic conditions (Fig. 1b). Interestingly, Plk4 transcript and protein levels post hypoxia treatment in the *Plk4^+/+^* MEFs were comparable to the levels normally found in *Plk4* heterozygous cells. Moreover, treated *Plk4* heterozygous MEFs displayed even further depleted Plk4 protein levels by approximately 10% compared to the untreated counterpart (Fig. 1c,d). This suggests that the *Plk4* promoter region may be targeted for methylation under hypoxic conditions. Next, we sought to determine whether the modification to the epigenetic marks that we observed were specific to *Plk4*, or if the other *Plks* were also undergoing a similar response. Interestingly, hypoxia treatment of wild-type MEFs resulted in hypermethylation of the *Plk1* promoter region (Fig. 1a) with a corresponding seven-fold decrease in transcript levels (Fig. 1e) and a 20% decrease in protein levels when compared to non-treated controls (Fig. 1f). Considering that *Plk1* was methylated prior to treatment in *Plk4^+/−^* MEFs, it was not surprising to see that there was no change in the methylation status of *Plk1* promoter with hypoxia (Fig. 1a). In contrast, there was a moderate increase in the corresponding *Plk1* transcripts (Fig. 1e). Examination of Plk1 protein levels in untreated *Plk4^+/−^* MEFs revealed almost 40% higher Plk1 levels compared to the wild type cells prior to treatment (Fig. 1f). Moreover, post-treatment, *Plk4^+/−^* MEFs showed approximately a 10% increase in Plk1 protein levels compared to the untreated (Fig. 1f). As a positive control, Hif1α transcript levels were assessed post treatment to ensure the cells were responding to hypoxic conditions (Fig. 1g).

Previous research has shown that p53 is both necessary and sufficient in transcriptionally repressing Plk1 [26]. In a regenerating liver model, *Plk4* heterozygosity resulted in decreased p53 protein levels and activity compared to the wild-type model as evidenced by decreased p21 levels and phosphorylated Ser15 on p53 [2], suggesting that *Plk4* heterozygosity is insufficient for proper p53 activation. This also suggests a model in which *Plk1* expression in wild-type MEFs exposed to hypoxia is in part regulated by promoter methylation, resulting in repression of transcription and lower protein levels. The different response for *Plk1* in *Plk4^+/−^* MEFs, is likely related to the fact that *Plk4^+/−^* MEFs display increased genomic instability along with a lack of active p53 during stress [2]. Thus, the normal regulatory mechanisms necessary to down-regulate Plk1 protein levels are, in part, absent. This combination of lower Plk4 and increased Plk1 likely results in promoting the cellular transition through G2/M, and further propagating genomic instability and aneuploidy resulting in DNA damage caused by *Plk4* haploinsufficiency [2], a contributing factor to tumourigenesis. It also further suggests that Plk4 needs to be at normal levels in order to maintain appropriate Plk1 levels.

### ROS-induced epigenetic downregulation of the *Plks* in MEFs

**Figure 2:**
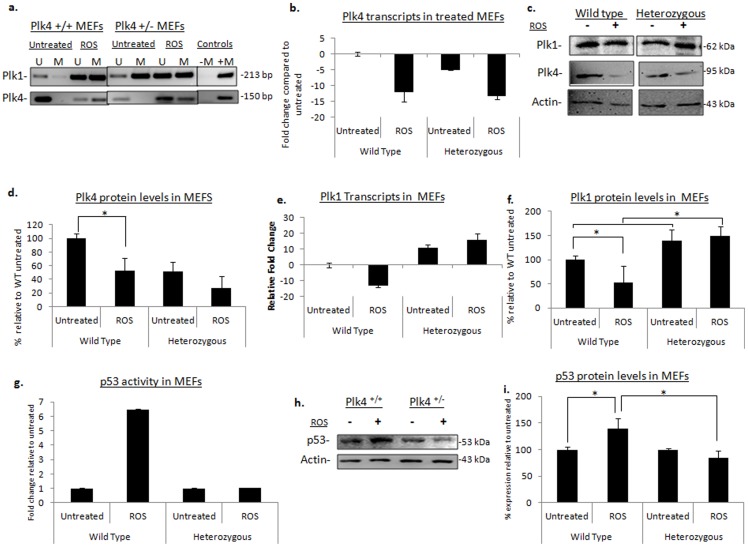
Modification of *plk1* and *plk4* epigenetic marks with ROS exposure in MEFs. (a) MSP analysis shows the promoter methylation of *plk1* and *plk4* pre- and post-ROS treatment; U = unmethylated, M = methylated. Fully methylated NIH 3T3 DNA was used as a positive control (+M), no template was added to the negative control (−M). (b) Plk4 transcript levels determined by qPCR. All transcripts were normalized to the wild type untreated control. All qPCR data is representative of the mean value of three independent experiments and error bars represent +/− SD. (c) Plk1 and plk4 protein levels examined *via* Western blot analysis, actin was used as a loading control. (−) represents the lysates from untreated cells, (+) lysates from cells grown in the presence of ROS (d) Plk4 protein expression levels determined by densitometry. All densitometry data is representative of three independent experiments and the error bars represent +/− SD. * denotes significance with a p<0.05. (e) Plk1 transcripts of cells treated with ROS, the transcripts were normalized to the respective untreated samples. (f) The relative plk1 protein levels post treatment was normalized to the wild-type untreated samples. Levels determined by densitometric analysis of Western blot images. (g) An ELISA-based p53 activity assay. Relative activity was determined by normalizing values to the untreated samples. This data represents the mean value obtained over three independent experiments and error bars denote the +/− SD. (h) p53 protein levels in MEFs post treatment as determined by Western blot analysis. (i) Densitometry was performed on three independent experiments and all data has been normalized to the respective untreated. The mean expression is presented with error bars denoting +/− SD. * denotes significance with a p<0.05.

Oxidative stress in the microenvironment is not limited to hypoxic conditions. Oxidative stress can also be caused by an increase in free radicals producing reactive oxygen species (ROS). Furthermore, ROS have been shown to promote tumourigenesis through several biological processes which include cell proliferation, metastasis, and evasion of apoptosis [27]. Exposure of cells to high levels of ROS have also been implicated in the hypermethylation of tumour suppressor genes such as runt-related transcription factor 3 (*RUNX3*) [28]. Moreover, ROS exposure, as a result of hydrogen peroxide treatment, has been shown to recruit DNA methyltransferases (DNMT) complexes to areas in the genome that are CG-rich, which could include the CpG islands upstream of the *Plk* promoter regions [29]. Additionally, in our previous work, we demonstrated that wild type MEFs that were chronically exposed to ethanol (EtOH) treatment, displayed a hypermethylated *Plk4* promoter region resulting in a phenotype that resembles that seen in *Plk4^+/−^* cells with multi-nucleation and multiple-centrosome formation [10]. Inherent to ethanol metabolism is the production of high levels of ROS [30] therefore, suggesting that ROS may also impact *Plk* promoter methylation. In order to examine whether *Plk1* and *Plk4* epigenetic marks were susceptible to modification as a result of high levels of ROS, we subjected *Plk4^+/+^* and *Plk4^+/−^* MEFs to reactive oxygen species (ROS) by exposing them to hydrogen peroxide (H_2_O_2_) at a 200 um dose for a period of 18 hours. This level of ROS is known to induce DNA damage and p53 activity [31]. Methylation specific PCR (MSP) revealed that the *Plk4* promoter became hypermethylated in the presence of ROS (Fig. 2a). Both *Plk4^+/+^* and *Plk4^+/−^* MEFs displayed a decrease in *Plk4* transcripts of more than 10-fold (Fig. 2b) and subsequent Western blot analysis revealed a significant decrease in Plk4 protein in both MEF genotypes by approximately 50% (p<0.05) relative to the untreated cells (Fig. 2c,d). These results are similar to what we observed under hypoxic conditions, and suggest that as part of the stress and DNA damage response, *Plk1 and Plk4* may normally become downregulated *via* promoter methylation likely in order to arrest cell division. It is noted previous work by Ko et al. revealed that low levels of Plk4 results in a delay in cell cycle progression [2], and we have shown that lower levels of *Plk4* results in cells aggregating at the G2/M transition of the cell cycle [32].

*Plk1* promoter methylation and levels in *Plk4^+/+^* MEFs were responsive to increased ROS in a similar manner to that seen with hypoxia, in which *Plk1* was downregulated (Fig. 2a). *Plk1* transcripts were decreased by approximately 12-fold, which was reflective of the promoter hypermethylation (Fig. 2e). This was correlated with visibly reduced protein levels post ROS exposure by almost 40% (Fig. 2c,f). Although there appeared to be no visible change at the promoter region *via* MSP analysis, *Plk1* transcripts were elevated in the heterozygous MEFs in the presence of ROS with transcripts almost 15-fold higher compared to the untreated (Fig. 2a,e). Moreover, Plk1 protein expression levels were also 10% higher in ROS-treated heterozygous MEFs compared to the untreated counterparts and 100% higher compared to the treated wild-type MEFs (p<0.05) (Fig. 2c,f). In contrast to *Plk4 and Plk1*, *Plk2* promoter methylation as well as Plk2 and Plk3 protein levels displayed no detectable changes in either cell type in response to hypoxia and upon exposure to reactive oxygen species (Figure S1a,b). Note that, we did not examine *Plk3* promoter methylation as the gene in mouse lacks CpG islands.

The experimental results observed for *Plk1* and *Plk4* epigenetic regulation in MEFs as a response to ROS were similar to those obtained under hypoxic conditions, suggesting that an adequate response to stress and the DNA damage may be impaired in *Plk4^+/−^* MEFs and that lower Plk4 protein levels have an indirect impact on the epigenetic regulation of *Plk1*. This model is supported by the observations that upon DNA damage, p53 is activated and subsequently represses Plk1 [33], [34]. Previous work has determined that p53 interacts with and is a substrate of Plk4; and in the *Plk4^+/−^* mouse model, partial hepatectomy failed to activate p53 within the first 24 hours post-surgery, unlike the wild-type counterparts which displayed p53 activation almost immediately [2], [35].

Given these observations, it was therefore of interest to determine whether p53 was activated in *Plk4^+/−^* MEFs post ROS treatment. We performed an ELISA-based p53 activity assay with MEF nuclear extracts post H_2_O_2_ treatment. *Plk4^+/+^* cells had an increase in p53 protein levels by almost 50% and an increase in p53 activity by almost 6-fold relative to the untreated cells (Fig. 2g–i). Unexpectedly, in *Plk4^+/−^* MEFs, p53 activity was not elevated, but was comparable to the untreated counterparts (Fig. 2g). This corresponded to the lack of a significant change in p53 protein levels for the *Plk4* ^+/−^ MEFs (Fig. 2h,i). Our observations suggest that *Plk4* heterozygosity and the subsequent low Plk4 protein levels are insufficient to activate p53 during genotoxic stress caused by ROS, resulting in an upregulation in the pro-mitotic protein, Plk1. Interestingly, in our previous examination of HCC in *Plk4^+/−^* mice, we also observed elevated Plk1 protein in tumours, but not in normal liver tissue [10]. Human studies have found that loss of heterozygosity for *PLK4* occurs in 45–60% of HCC cases examined together with an increase in Plk1 protein levels [2], [9]. *PLK4* LOH may be an early event in the progression to carcinogenesis. Here we show that a combinatorial effect of *Plk4* heterozygosity, together with micro-environmental stressors such as hypoxia and ROS, result in the upregulation of Plk1.

### Promoter methylation of the *Plks* in HCC tumour cells

**Figure 3:**
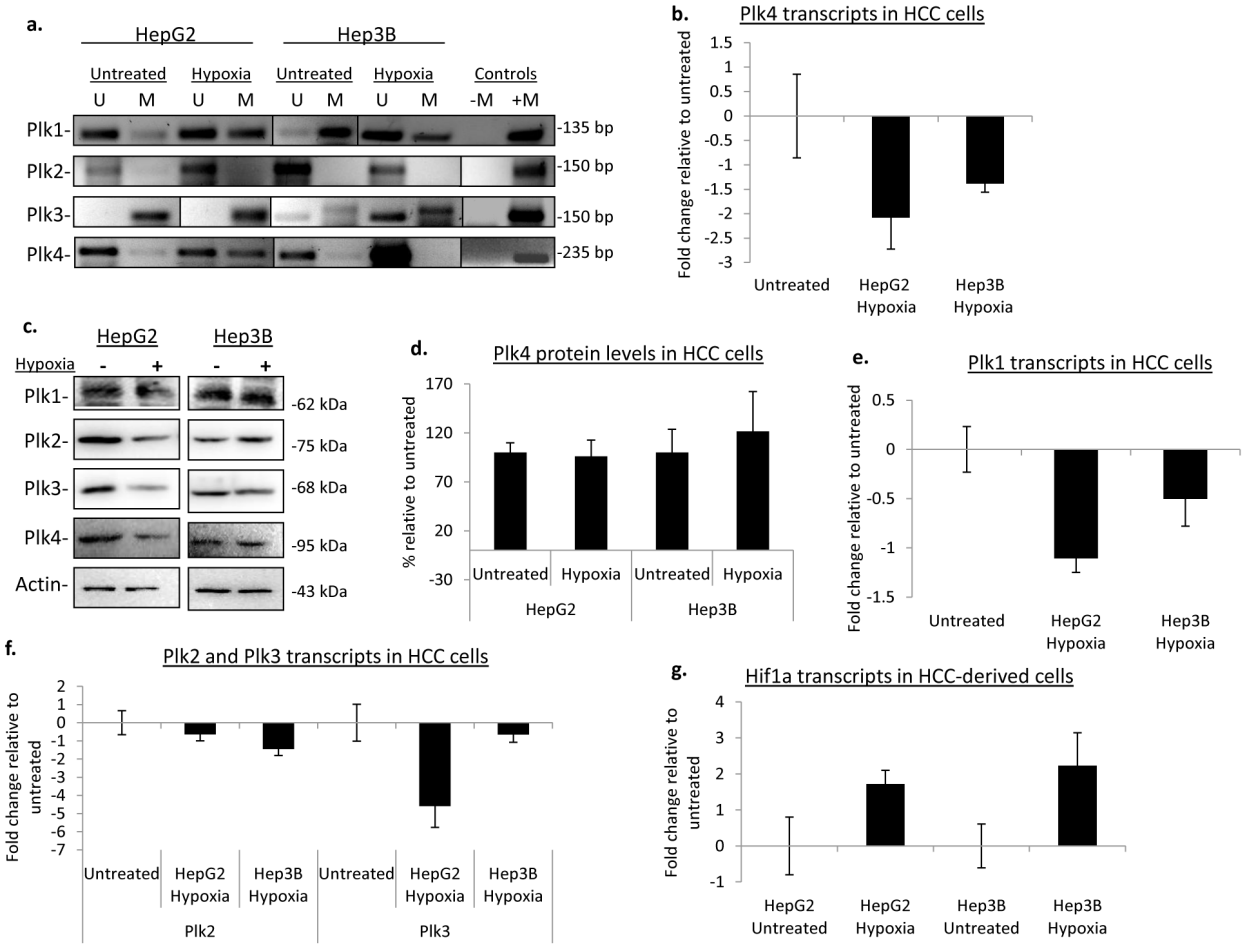
Hypoxia-induced modification of *PLK* promoter methylation in HCC cells. (a) Promoter methylation status of the plks examined in HCC-derived cells HepG2 and Hep3B; U = unmethylated, M = methylated. Fully methylated HeLa DNA was used as a positive control (+M), no template was added to the negative control (−M). (b) Post hypoxia, PLK4 transcripts were assessed *via* qPCR in RNA extracted from HCC cells. All qPCR data is representative of the mean value of three independent experiments and error bars represent +/− SD. (c) PLK protein levels were examined post treatment from whole cell lysates. Actin was used as a loading control. (−) represents lysates from untreated cells, (+) lysates from cells grown in the presence of hypoxia. (d) Quantification of protein levels using densitometry. Levels have been normalized to the respective untreated controls. Data is representative of the mean value of three independent experiments and error bars represent +/− SD. (e) The fold change of PLK1 transcripts as determined by qPCR. Values normalized to the respective untreated sample. (f) PLK2 and PLK3 analyzed and fold changed determine by normalization to the respective untreated samples. (g) Hif1α transcripts post hypoxia were determine by real-time PCR using a Taqman probe.

Li et al. 2005 demonstrated that PLK4 mRNA is regulated in a p53-dependent manner in lung carcinoma cells and osteosarcoma-derived cells exposed to etoposide [36]. The levels of *PLK4* transcripts were most affected at 6 and 24 hours post treatment [36]. Thus, p53 plays a role in the transcriptional downregulation of *PLK4* through histone deacetylation upon exposure to DNA damaging agents [36]. Recently, Nakamura et al. also showed that sustained genotoxic stress *via* etoposide and UV resulted in the attenuation of PLK4 in a p53-dependent manner [37]. In addition, p53 is known to be an important player in the epigenetic downregulation of another tumour suppressor, ras-associated domain family 1 (*RASSF1A*), by directly binding to the promoter of *RASSF1A* and recruiting DNA methyltransferase 1(DNMT1) along with accessory proteins to the promoter region [38]. Moreover, p53 interacts and cooperate with DNMT1 in the methylation of the PLK4 target, CDC25C, in the presence of DNA damage [39] and also interacts with DNMT3a, which is responsible for *de novo* methylation [40]. This suggests that p53 likely also regulates the *Plks* through an epigenetic mechanism. We were therefore interested in determining whether the promoter methylation of the *Plks*, which we observed in MEFs under hypoxia and ROS treatment, was dependent on the presence or absence of p53. We employed the hepatocellular carcinoma (HCC) derived cell lines, HepG2 and Hep3B to answer this question. Both HepG2 cells and Hep3B cells exhibited an increase in *PLK4* promoter methylation post hypoxia (Fig. 3a). In the case of HepG2 cells there was an increase in the detectable level of methylation accompanied with a corresponding 2-fold decrease in *PLK4* transcripts (Fig. 3b) compared to the untreated as well as a 5% decrease in protein levels (Fig. 3c,d). For Hep3B cells, under hypoxic conditions, the increase in promoter methylation did not translate into significant changes at transcript and proteins levels (Fig. 3c,d). In this case, protein levels of PLK4 did not show a significant difference, although transcript levels were slightly decreased (Fig. 3c,d). As HepG2 cells contain a functional p53 whereas as Hep3B cells lack a functional p53 [41], these results once again suggest the involvement of p53 in the epigenetic regulation of *PLK4*.

Likewise, for *PLK1*, the change in methylation status was similar to that seen with hypoxia treatment in MEFs. Before treatment, HepG2 cells displayed some methylation for the *PLK1* promoter (Fig. 3a). Post hypoxia, the *PLK1* promoter region became hypermethylated (Fig. 3a). In addition, transcript levels were decreased by almost 2.5-fold (Fig. 3e) and accompanied by a slight decrease in protein levels (Fig. 3c). Hep3B cells, on the other hand, showed no distinct change in the methylation status of *PLK1* promoter region compared to the untreated (Fig. 3a). Moreover, PLK1 transcript and protein levels in treated Hep3B cells were not significantly impacted by hypoxia treatment (Fig. 3c,e).

Human *PLK3*, unlike its murine homolog has two CpG islands in its promoter region. We used two sets of primers in order to assay for any changes in methylation status for *PLK3*. With both, MSP published primers based on the first 200 base pairs of the upstream CpG island [1] and an additional set of MSP primers downstream, we detected no overt change in promoter methylation for *PLK3* in either HepG2 or Hep3B cells (Fig. 3a). This suggests that the regulation of *PLK3* under hypoxic conditions is not p53 dependent and is likely not regulated by an epigenetic mechanism in this context.

Likewise, for *PLK2*, there was no dramatic change in promoter methylation, for either HepG2 and Hep3B cell lines. This indicates that *PLK2* and *PLK3* do not undergo aberrant changes to their promoter methylation in response to hypoxia.

As an experimental control, we assessed the transcript levels of HIF1α to determine whether these cells were responding to hypoxic stress under the same hypoxic conditions as used with the MEFs. With hypoxia, *HIF1α* transcripts were elevated by more than 1.5 times in both cell lines (Fig. 1g), indicating that the cells were indeed responding to low oxygen levels and the change in *HIF1α* transcript levels were similar to previously reported hypoxia treatments in HCC cells [42].

### *Plk* promoter methylation in HCC with ROS treatment

**Figure 4:**
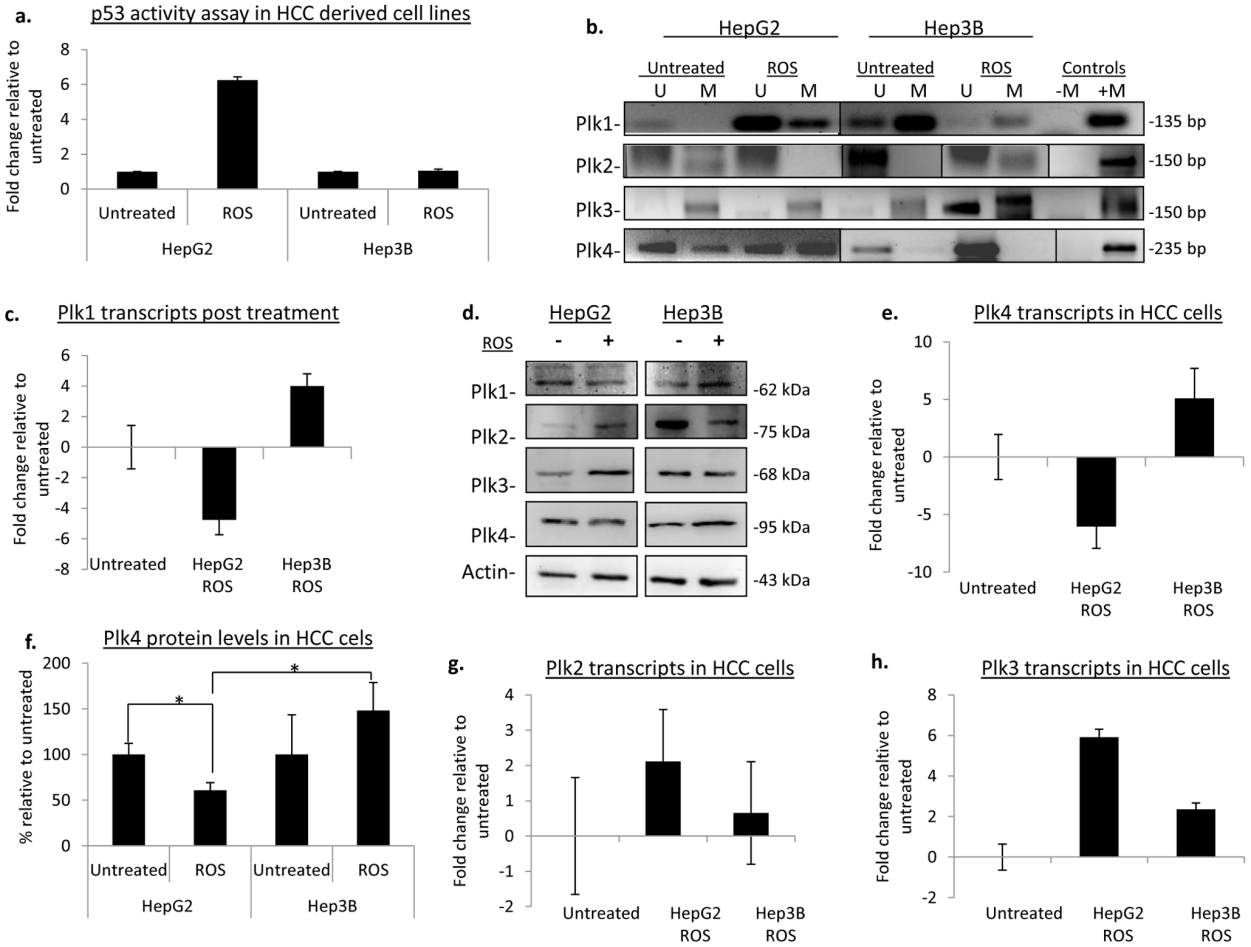
Modification of *PLK* promoter methylation marks in HCC cells exposed to ROS. (a) A p53 activity assay was performed to confirm activation of p53 with genotoxic stress caused by ROS. The percent activity is the average of three independent experiments with error bars representing the +/− SD. (b) MSP analysis of *plk* promoter methylation; U = unmethylated, M = methylated. Fully methylated HeLa DNA was used as a positive control (+M), no template was added to the negative control (−M). (c) Plk1 transcript levels were examined and normalized to the respective untreated samples. All qPCR data is representative of the mean value of three independent experiments and are normalized to the untreated samples. Error bars represent +/− SD. (d) Western blot analysis of PLK protein levels. Actin was used as a loading control. (−) represents the lysates from untreated cells, (+) lysates from cells were grown in the presence of ROS. (e) The fold change in plk4 transcripts from cells exposed to ROS. (f) Quantification of PLK4 protein levels. Data is representative of three independent experiments and the error bars represent +/− SD. * denotes significance with a p<0.05. (g,h) PLK2 and PLK3 change in transcripts as determined by real time PCR.

HepG2 and Hep3B were cultured in the presence of hydrogen peroxide at a concentration of 200 um and activation of p53 by ROS was confirmed via an ELISA-based p53 activity assay and Western blot analysis. As expected, we found a 6-fold increase in p53 activity in HepG2 cells in the presence of ROS, while no change in activity was detected for Hep3B (Fig. 4a). The increase in activity also corresponded to an increase in p53 protein levels in HepG2 cells, while in agreement with Hep3B p53 status, no p53 protein was detected in Hep3B cells (Figure S1c). *PLK1* became hypermethylated in HepG2 post ROS exposure, while in Hep3B the level of detectable methylation decreased in comparison to that initially present in untreated cells (Fig. 4b). Subsequent examination of the transcript and protein expression for PLK1 were correlated with their respective promoter methylation status. Specifically, in HepG2, PLK1 transcripts and protein were significantly reduced, whereas in Hep3B, *PLK1* transcripts were almost 4-fold higher compared to the untreated control and protein expression was also elevated (Fig. 4c,d). Here we show that *PLK1* downregulation in response to DNA damage in p53-wild type cells is also accompanied by promoter hypermethylation and this hypermethylation can be induced by ROS whereas the opposite scenario is observed for the p53 null cells.

*PLK4* promoter methylation patterns also paralleled what we have observed with *PLK1*, where HepG2 had a qualitative gain in *PLK4* promoter methylation (Fig. 4b) accompanied by a 6-fold decrease in transcripts and a 40% decrease in protein expression (Fig. 4d–f). This is in direct opposition to what we observed in Hep3B cells, which had no observable gain of methylation for *PLK4*, but more importantly, there was an increase in transcripts and protein by 5-fold and 30% respectively compared to the untreated cells (fig. 4d,e).

This data indicates that *PLK1* and *PLK4* promoter methylation is p53-dependent and that ROS may play an important role in the regulation of both of these genes. This correlates with recent work by Nakamura et al. which determined that under stress and DNA damage in colorectal cells, PLK4 is initially activated, but its expression is abrogated over time in p53-wild type cells followed by an increase in p53 levels. In p53-null cells, PLK4 protein levels persisted over the same period of time [37].

Previous examination of *PLK2* expression has shown that it can be induced by p53 during DNA damage and stress *via* p53 directly biding to its consensus sequence within the *PLK2* promoter [43], [44]. More recently, *PLK2* transcript levels have been used as predictors in determining the genotoxicity of potential hepatocarcinogens [45]. So, it was not surprising to see that post ROS treatment of HepG2 cells, *PLK2* lost promoter methylation (Fig. 4b) along with a 2-fold increase in *PLK2* transcript (Fig. 4g) and protein levels (Fig. 4d). In Hep3B cells, *PLK2* displayed a gain of methylation at its promoter region after ROS exposure (Fig. 4b), correlated with decreased protein levels, suggesting that in the absence of p53, the *PLK2* promoter region becomes hypermethylated in HCC in the presence of ROS (Fig. 4d).

PLK3 activity is also known to become upregulated in the presence of H_2_O_2_. This increase in activity leads to the phosphorylation of p53 at serine 20 in human fibroblast cells [31]. Therefore, we would expect PLK3 levels to increase in response to ROS treatment. Although *PLK3* promoter methylation remained largely unchanged between the untreated and the ROS exposed cells (Fig. 4b), *PLK3* transcripts (Fig. 4h) and protein levels (Fig. 4d) were elevated in ROS treated HepG2 cells. However, in the absence of p53, *PLK3* transcripts and protein levels were not significantly changed with ROS treatment (Fig. 4h,d).

Here we show that in HCC cells, *PLKs* 1,2, and 4 become epigenetically modified in the presence of ROS, and that this regulation is in part, p53 dependent. Moreover, in Hep3B cells, which lack p53, the upregulation of the *PLKs* needed for DNA damage repair, PLK2 and PLK3, are impaired in the presence of ROS. This is also accompanied by an increase in PLK1 and PLK4 in p53 null cells. In the clinical setting, PLK1 and PLK4 have been found to be jointly upregulated in colorectal cancers compared to the normal mucosa in almost 80% of the cases examined [4]. Furthermore, upregulation of PLK4 leads to centrosome amplification and multipolar spindle formation resulting in aneuploidy, which is a signature of many solid tumours [46]. In addition, it is important to note that more than 50% of colorectal cancers harbour p53 mutations [47].

### *Plk* promoter methylation in osteosarcoma-derived cells

**Figure 5:**
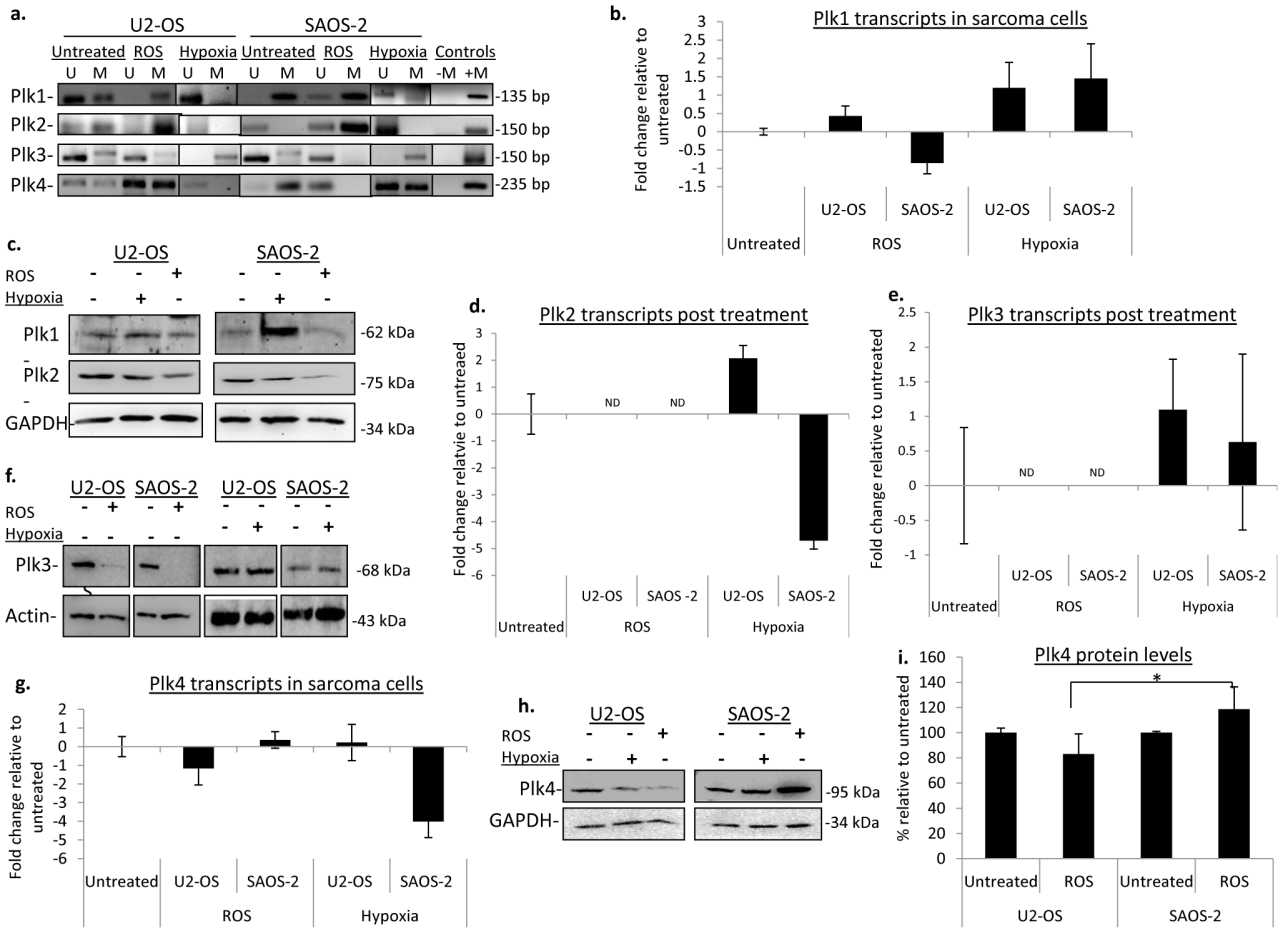
Examination of *PLK* promoter methylation in sarcoma-derived cells grown in the presence of oxidative stress. (a) *PLK* promoter methylation was determined by methylation-specific PCR; U = unmethylated, M = methylated. Fully methylated HeLa DNA was used as a positive control (+M), no template was added to the negative control (−M). (b) Fold change in plk1 transcripts. All qPCR values have been normalized to the respective untreated samples. Here the mean value of three independent experiments are depicted with error bars representing the +/− SD. (c) PLK1 and PLK2 protein levels in U2-OS and SAOS-2 cells treated with hypoxia and ROS. GAPDH was used as a loading control. (−) indicates lysates extracted from untreated samples, (+) represents lysates extracted from cells exposed to either hypoxia or ROS. (d,e) PLK2 and PLK3 transcripts as determined by qPCR. ND = not detectable. (g) Transcript changes for PLK4 in cells exposed to ROS and hypoxia. (h) PLK4 protein levels in sarcoma cells treated with hypoxia and ROS (+) compared to the untreated counterpart (−). GAPDH was used as a loading control. (i) PLK4 protein levels quantified with densitometry analysis of the Western blot images. The histogram is representative of the mean from three independent experiments with error bars showing the +/− SD. * denotes significance with a p<0.05.

These results raised the question whether these modifications were a general phenomenon or were these epigenetic modifications specific to tissue or cell type? Previous literature suggested that certain gene-signatures that are found in HCC cells are not found in other cell types such as colon carcinomas [42]. We chose to replicate our experiments with hypoxic conditions and in the presence of ROS using osteosarcoma derived cells within the same p53 context. We employed the p53-wild type cells U2-OS and the p53 null cells Saos-2 [41]. First, we examined the promoter methylation and expression of the *PLKs* in the sarcoma-derived cells under hypoxic conditions. Interestingly, in osteosarcoma cells, *PLK1* promoter regions became hypomethylated in both U2-OS and Saos-2 cells (Fig. 5a) followed by upregulation of the accompanying transcripts and protein levels compared to the untreated cells (Fig. 5b,c). This suggests that hypoxia-induced modifications to the promoter methylation of *PLK1* in the above mentioned cell lines is not p53 dependent. Conversely, when examining the *PLK2* promoter methylation under hypoxic conditions, U2-OS cells displayed a loss of promoter methylation (Fig. 5a) followed an almost 2-fold increase in transcripts (Fig. 5d), while a only a slight change in protein level was observed (Fig. 5c); Saos-2 cells on the other hand, had no distinct change in promoter methylation (Fig. 5a), however, qPCR analysis revealed a decrease in *PLK2* transcripts by almost 5-fold resulting in a slight decrease in protein (Fig. 5c,d). A study by Matthew et al. revealed that PLK2 has an active and p53-dependent role in the cellular response to hypoxia by indirectly restraining the mTOR signaling pathway during hypoxia, so it was expected that we would see an increase in PLK2 in U2-OS and not Saos-2 [48]. When examining the remaining *PLKs*, *PLK3*'s promoter region did not appear to change in response to hypoxia, in either cell type and transcript and protein levels did not differ from the untreated (Fig. 5a,e,f), similar to what we have seen in the MEFs and HCC cells. In Saos-2 cells, the *PLK4* promoter region became hypermethylated in the presence of hypoxia (Fig. 5a) followed by a decrease in *PLK4* transcripts by nearly 4-fold compared to the untreated (Fig. 5g), which resulted in a moderate decrease in protein levels (Fig. 5h). In U2-OS, the *PLK4* promoter region was initially methylated prior to treatment, but with hypoxia treatment, there was a loss of detectable methylation, though this did not translate into significant changes at the transcript or protein levels (Fig. 5a,g–i). The examination of sarcoma cells illustrates that hypoxia can differentially impact the *PLK* promoter methylation patterns between cell types, and that p53 may not have the same impact on the epigenetic regulation of the *PLKs* in all cells. *HIF1a* transcript levels were examined and were found to be elevated by 1.5–2 fold in both cell types (Figure S2a).

ROS treatment of sarcoma cells resulted in very different pattern of methylation than that seen in HCC cell lines. Confirmation of ROS-induced increased in p53 activity was carried out *via* Western blot analysis and with a p53 activity assay, which showed an increase in p53 activity in U2-OS cells by almost 9-fold, whereas no change was detected with SAOS-2 (Figure S2b,c). Unlike HCC cells, in both osteosarcoma cell lines, *PLK2* became hypermethylated (fig. 5a) accompanied by undetectable transcripts and significantly decreased protein levels (Fig. 5c,d). Although *PLK3* promoter methylation did not increase with treatment, transcripts and protein levels were also undetectable in either cell type (Fig. 5e,f). This suggests that *PLK2* and *PLK3* are differentially regulated in osteosarcoma cell lines compared to HCC cell lines. The *PLK1* promoter region also did not display a change in promoter methylation, remaining hypermethylated in both cell lines similar to our observations in HCC and MEFs (Fig. 5a). Real-time PCR did reveal a slight decrease in *PLK1* transcripts (Fig. 5b) and protein levels in SAOS-2 cells, but not in U2-OS cells (Fig. 5c). However, when examining *PLK4*, we noticed a dramatic loss of promoter methylation in Saos-2 cells in response to ROS, but not in U2-OS cells (Fig. 5a). Along with promoter hypomethylation in Saos-2 there was a minor increase in transcripts (Fig. 5a, g). PLK4 protein levels were also elevated in treated Saos-2 cells by more than a 10%; whereas U2-OS cells displayed a decrease in PLK4 protein by almost 20% compared to the untreated, similar to the response observed in HCC cells (Fig. 5h,i). This suggests that regardless of cell type, *PLK4* continues to be sensitive to ROS-induced promoter hypermethylation within a functional p53 context.

### Global methylation and DNMT levels

**Figure 6:**
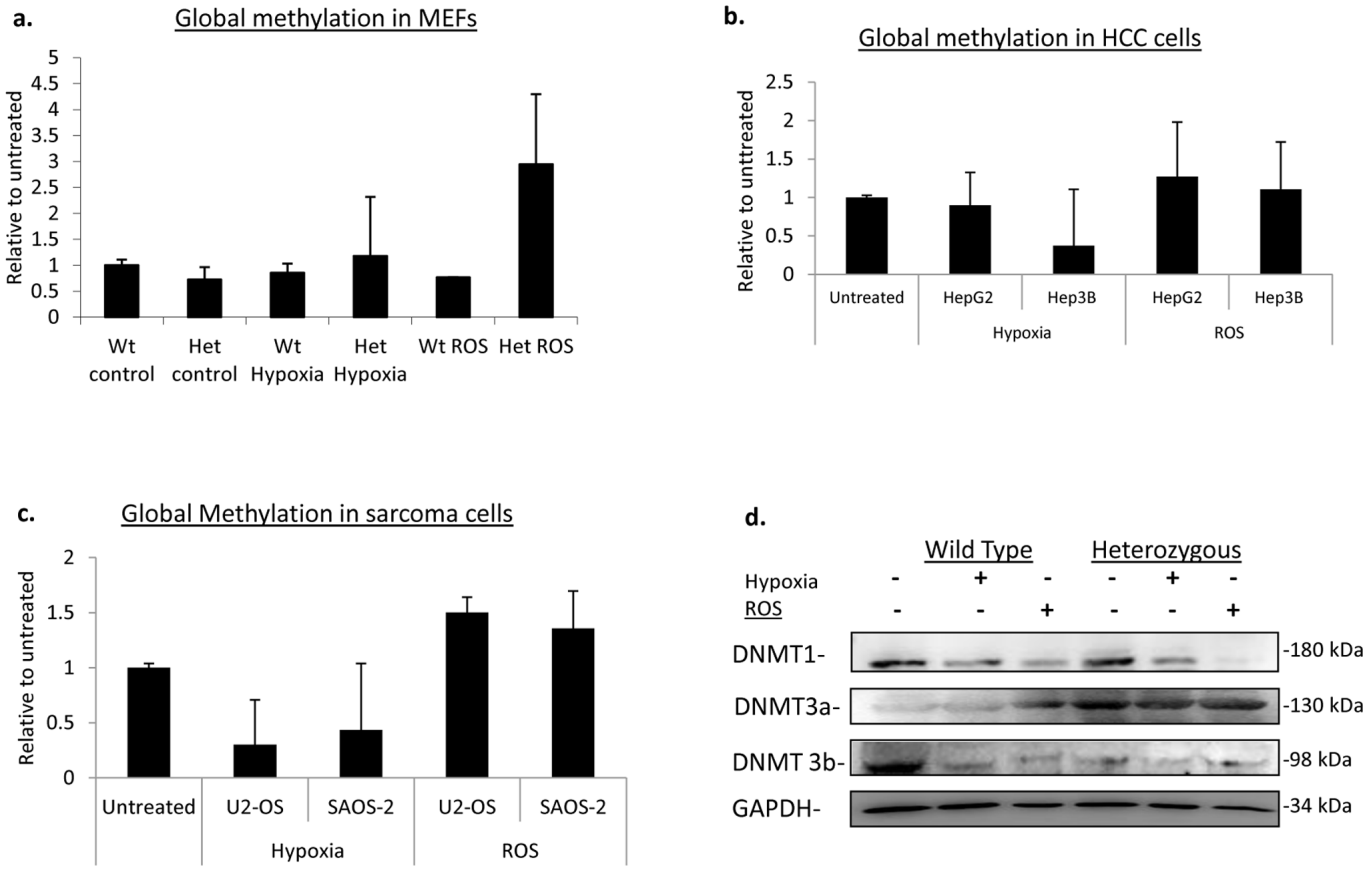
Analysis of global methylation in MEFs, HCC and osteosarcoma cells and DNMTs levels in MEFs. An ELISA-based global methylation assay was performed to determine changes in global methylation levels due to oxidative stress as a result of hypoxia and ROS exposure. The histograms are representative of three independent experiments and the error bars depict the +/− SD. (a) In MEFs the values have been normalized to the untreated wild-type cells. (b,c) The values have been normalized to the respective untreated samples. (d) Western blot analysis was used to determine the levels of the DNMTs from whole cell lysates extracted from untreated (−) and treated (+) MEF cells.

In general, cells exposed to oxidative stress also experience shifts in global methylation patterns that can be associated with modifications to local methylation patterns at gene promoter regions [49], [50]. As part of our epigenetic analysis of the *Plks*, we wanted to determine if the modifications we observed at *Plk*-specific promoter regions were associated with a general increase in global methylation and whether any change varied between p53 wild type and p53 null cells. Here we examined the whole genome methylation of DNA from cells subjected to either ROS or hypoxia treatment. With hypoxia, both *Plk4* wild type and heterozygous MEFs had a slight decrease in global methylation compared to the untreated samples by approximately 15% (Fig. 6a). This is similar to what Shahrzad et al. demonstrated in melanoma cells, under anoxia, global methylation decreased between 15–20% [49]. We also observed a similar trend with HCC and osteosarcoma cells, with a 15–40% decrease in global methylation (Fig. 6b,c). There was little difference in global methylation between the hypoxia treated p53 wild type and p53 null cells although, in three independent experiments, Hep3B cells displayed a greater loss of global methylation in comparison to HepG2 (Fig. 6b). DNA methylation is maintained by DNA methyltransferases (DNMTs) which are enzymes that catalyze the transfer of methyl groups to cytosines which are 5′ to guanine [51]. DNMT1 is responsible for maintenance methylation during replication, and DNMT3a and DNMT3b drive *de novo* methylation [51]. It was therefore of interest to determine whether the changes in global methylation were also accompanied by differences in protein levels of the DNMTs.

DNMT1 and 3b protein levels have both been shown to become downregulated with hypoxia along with a decrease in DNMT activity which would lead to an overall decrease in global methylation marks [52]. We examined the levels of the DNMT's in both wild type and *Plk4* heterozygous MEFs and found that this was also the case, where DNMT1 and DNMT3b protein levels decreased with hypoxia (Fig. 6d). When examining DNMT3a, protein levels were elevated in *Plk4* heterozygous MEFs prior to treatment and remained elevated post hypoxia treatment, but the wild type MEFs did not display this change in DNMT3a levels (Fig. 6d). It was previously reported that p53 wild type and p53 null colorectal cells, post hypoxia exposure, have increased transcript levels of DNMT3a, with a greater increase observed in p53 null colorectal cells [52]. Also, in an *in vivo* study done by Park et al., a p53 heterozygous and null mouse model revealed elevated levels of DNMT3a compared to the wild type littermates prior to any tumour development [53]. This suggests that DNMT3a is deregulated in *Plk4^+/−^* MEFs in a manner similar to that seen in p53 null cells. This also correlates to the decrease in p53 activity that we have observed in *Plk4^+/−^* MEFs and re-enforces the importance of the Plk4-p53 relationship and interaction axis.

ROS treated *Plk4^+/−^* MEFs also displayed an increase in global methylation (Fig. 6a), similar to what we observed in the HCC and osteosarcoma cancer cells (Fig. 6b,c). This was in contrast to global methylation levels in the *Plk4* wild type MEFs which decreased with ROS (Fig. 6a). This once again suggests that *Plk4* heterozygosity results in deregulation of the response to oxidative stress.

**Figure 7:**
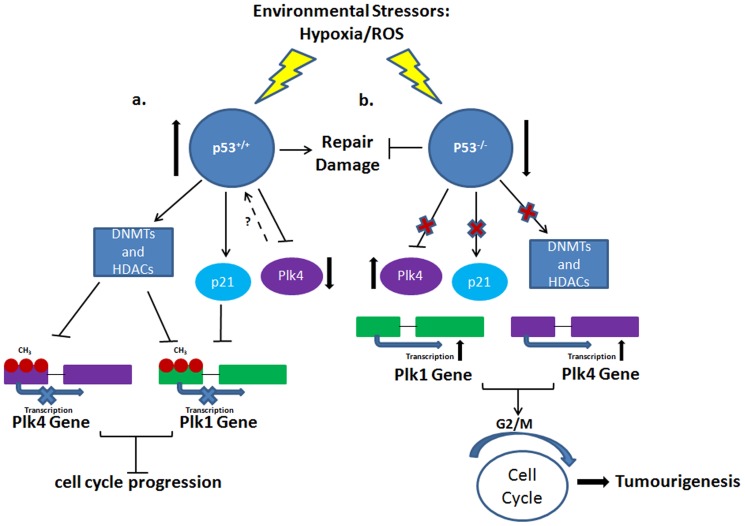
A potential role for p53 in the silencing of the *PLKs* as a result of oxidative stress. Previous data has established that p53 can regulate both PLK1 and PLK4 expression through protein-protein interactions. Here we have incorporated our observations into the known mechanisms of the p53-PLK regulatory axis (a.) Our data suggests that when oxidative stress upregulates p53 activity, this can lead to downstream effects that can potentially induce the epigenetic silencing of the *PLKs*. In wild type p53 cells, these mechanisms can include the recruitment and/or collaboration with epigenetic modifiers such as DNMT1, DNMT3a or histone deacetylases (HDACs). (b) However, oxidative stress in the absence of p53, these vital inhibitory interactions carried out through the p53 pathway are abolished. PLK1 and PLK4 expression thus carries on unhindered, potentially pushing the cell through the G2/M transition point with unrepaired DNA damage, resulting in genomic instability and aneuploidy, both of which are hallmarks of cancer.

The contributions to tumourigenesis are complex and multi-factorial. Oxidative stress has been acknowledged as one such contributor in the path to carcinogenesis. Previous studies have shown that the *PLKs* are subject to regulation through post-translational modifications [54], [55]. Our observations here show that the *Plks*, whose proper regulation is essential for cell cycle fidelity, become deregulated in the presence of both hypoxia and ROS through epigenetic modifications to their respective promoter regions. However, the deregulation that we have observed is cell-specific, resulting in methylation patterns that are similar, like those between MEFs and HCC, and patterns that differ like those observed in sarcoma-derived cells. The promoter methylation of *PLK4* is also correlated with the status of p53 in the cell. *Plk4* haploinsufficiency, together with oxidative stress-induced epigenetic deregulation can inadvertently lead to the upregulation of *Plk1*. Based on our observation and the current literature, we propose a model in which p53 likely leads to downregulation of transcription for *PLK1* and *PLK4* in the presence of cellular stress by either recruiting or cooperating with DNMT1, DNMT3a and/or histone deacetylases (HDACs); this leads to an increase in promoter hypermethylation and hence changes in expression [36]–[40] (Fig. 7a). In the absence of p53, cellular stress would lead to the upregulation of pro-mitotic PLKs (PLK1 and PLK4) resulting in a push through the G2/M checkpoint that would contribute to genomic instability and tumourigenesis (Fig. 7b)

The methylation status of the *PLKs* could also be used as an indicator of oxidative stress at the cellular level. These modifications to *PLK* epigenetic marks may even be an early event in the multi-stage process leading to tumourigenesis, given that we have observed detectable changes 18 hours post-treatment. Furthermore, promoter hypermethylation of the *PLKs* is a common event in a variety of cancers, including blood neoplasms, hepatocellular carcinoma, and ovarian cancer. Aberrant promoter methylation, induced specifically *via* microenvironemtal cues, could be another contributor to carcinogenesis [1], [8]–[11]. Currently, PLK1 has been the most targeted PLK for drug development [56]–[58], however, promoter hypermethylation is a reversible phenomenon for which there are drugs already in clinical use [59] that could be used as prophylactic agents or could help reverse hypermethylation-induced downregulation of the remaining four tumour suppressing PLKs in combination with traditional therapies.

## Materials and Methods

### Ethics Statement

All animal procedures were carried out in accordance with animal care protocols approved by the University of Windsor Animal Care Committee under the guidelines of the Canadian Council on Animal Care.

### Tissue Culture

Mouse embryonic fibroblasts (MEFs) were harvested from *Plk4^+/+^* and *Plk4^+/−^* embryos at day 12.5 *post coitus*, as described in Ko et al, 2005 [2]. The procedure was carried out in accordance with animal care protocols approved by the University of Windsor Animal Care Committee under the guidelines of the Canadian Council on Animal Care. The MEFs were maintained in DMEM supplemented with 20% fetal bovine serum and 1% penicillin G sodium/streptomycin sulphate at 10,000 ug/mL, and 0.5% gentamycin 10 mg/mL. Cell lines were purchased from ATCC, U2-OS and Saos-2 cells were maintained in McCoy's media supplemented with 10% FBS. Hep3G2 and Hep3B cell lines were grown in MEM with 10% FBS. All the cell lines were kept in a 37°C incubator with 5% CO_2_. During hypoxic treatment, cells were grown in a hypoxia incubator chamber (STEMCELL Technologies Inc.) flooded with 2% CO_2_ at a rate of 10 L/min for 8 minutes then incubated for 18 hours at 37°C. Reactive oxygen species were generated using 200 um H_2_O_2_ for 18 hours; treated cells were grown in standard culture conditions.

### Methylation Specific PCR

DNA was extracted from cells prior and post treatment using ProK digestion buffer (0.5 mg/mL) followed by phenol chloroform extraction. Genomic DNA was subjected to bisulfite conversion as described in Herman et al. [60]. Post-bisulfite treatment, the DNA was purified using the Wizard mini DNA clean-up kit (Promega), desulfanated with NaOH and ethanol precipitated. MSP was performed with primers designed for individual *Plks* using the MethPrimer program [61]. For sequences please see Ward et al. [10]. Positive controls of fully methylated NIH 3T3 mouse DNA and HeLa human DNA (NEB) were also included in all experiments.

### Western blot analysis

Whole cell lysates were extracted using a lysis buffer (50 mM Tris-HCl pH 7.4, 150 mM NaCl, 1 mM EDTA, 0.5% Triton X) with an EDTA-free protease inhibitor cocktail (Bio Basics Inc.) 20 ug of total protein was used to perform Western blot analysis. Primary antibodies were purchased accordingly, anti-PLK2, anti-PLK3, and anti-DNMT3b (from Santa Cruz), anti-PLK1, anti-PLK4, anti-GAPDH, and anti-DNMT3A (from Cell Signalling), and anti-DNMT1 and anti-Actin (from Sigma). For secondary antibodies, anti-rabbit (from Cell Signalling) and anti-mouse HRP (from Sigma) were used. Bands were visualized by ECL (Thermo Scientific) and blots were acquired on an Alpha Innotech Multimage™ Light Cabinet and densitometry analysis was carried out using OptiQuant software Version 5.0.

### Real time PCR

RNA from treated cells was extracted using the RNeasy® mini kit (Qiagen). RNA extraction was performed according to manufacturer's protocols. Reverse transcription was carried out using the First Strand cDNA synthesis kit (Invitrogen) according to manufacturer's instructions. Real time PCRs were carried out on an ABI 7300 machine using Taqman gene expression probes for mouse *Plk1*, *Plk4*, and *HIF1α*; and human *PLK1-PLK4*, and *HIF1α* (Applied biosystems). GAPDH was used as an internal control in ROS qPCR, but not in hypoxia qPCR due to GAPDH transcripts also being affected by hypoxia treatment [62]. Hypoxia transcript values were normalized by addition of 100 ng of total cDNA to each reaction and ΔCT for treated samples were calculated from the untreated CT values as per [57]; additional calculations were performed according to the Taqman assay manual.

### p53 activity assay

The Human/Mouse Active p53 DuoSet® IC (R&D Systems) assay was carried out according to the manufacturer's protocol. Briefly, cells were grown to 80–90% confluence and the nuclear fraction of protein was extracted and sandwich ELISA assay was used to determine p53 activity.

### Global methylation assay

The global methylation of genomic DNA was determined by using 100 ng of ProK extracted gDNA in a sandwich ELISA colourimetric assay (Epigentek). The assay was carried out according to manufacturer's instructions.

### Statistical analysis

All Western blot analysis, transcript levels, and global methylation assays are represented as the mean +/− standard deviation. These data were evaluated using Statsoft Statistica software version 7.1 using One-way ANOVA analysis. Significance represents a p<0.05. All results are representative of three independent experiments.

## Supporting Information

Figure S1 **Assessment of plk2 and plk3 levels in treated MEFs and p53 levels in HCC cells.** (a) Methylation status of *Plk2* in treated MEFs was determined by MSP. (b) Western blot analysis of Plk2 and Plk3 protein in untreated (−) and treated (+) MEFs. GAPDH was used as a loading control. (c) p53 protein levels determine *via* Western blot analysis in untreated (−) and treated (+) HCC cells. GAPDH was used as a loading control.
(PPTX)

Figure S2 **Examination of Hif1α transcripts along with p53 levels and activity in treated osteosarcoma cells.** (a) Transcript levels of Hif1α were determined by qPCR and normalized to the respective untreated samples. The histogram is representative of the mean from three independent experiments with errors bars showing +/− SD. (b) p53 protein levels in untreated (−) and treated (+) U2-OS and SAOS-2 cells. (c) The activity of p53 pre- and post-treatment from nuclear extracts of osteosarcoma cells. The values were normalized the respective untreated samples. Error bars represent the +/− SD from three independent experiments.
(PPTX)

## References

[1] Syed N, Smith P, Sullivan A, Spender LC, Dyer M, et al (2006) Transcriptional silencing of Polo-like kinase 2 (SNK/PLK2) is a frequent event in B-cell malignancies. Blood 107: 250–256. 16160013 10.1182/blood-2005-03-1194

[2] Ko MA, Rosario CO, Hudson JW, Kulkarni S, Pollett A, et al (2005) Plk4 haploinsufficiency causes mitotic infidelity and carcinogenesis. Nat Genet 37: 883–888. 16025114 10.1038/ng1605

[3] Knecht R, Elez R, Oechler M, Solbach C, von Ilberg C, et al (1999) Prognostic significance of polo-like kinase (PLK) expression in squamous cell carcinomas of the head and neck. Cancer Res 59: 2794–2797. 10383133

[4] Macmillan JC, Hudson JW, Bull S, Dennis JW, Swallow CJ (2001) Comparative expression of the mitotic regulators SAK and PLK in colorectal cancer. Ann Surg Oncol 8: 729–740. 11597015 10.1007/s10434-001-0729-6

[5] Wang R, Song Y, Xu X, Wu Q, Liu C (2013) The expression of Nek7, FoxM1, and Plk1 in gallbladder cancer and their relationships to clinicopathologic features and survival. Clin Transl Oncol 15: 626–632. 23359173 10.1007/s12094-012-0978-9

[6] Takai N, Hamanaka R, Yoshimatsu J, Miyakawa I (2005) Polo-like kinases (Plks) and cancer. Oncogene 24: 287–291. 15640844 10.1038/sj.onc.1208272

[7] Liu L, Zhang CZ, Cai M, Fu J, Chen GG, et al (2012) Downregulation of polo-like kinase 4 in hepatocellular carcinoma associates with poor prognosis. PLoS One 7: e41293. 22829937 10.1371/journal.pone.0041293 PMC3400587

[8] Syed N, Coley HM, Sehouli J, Koensgen D, Mustea A, et al (2011) Polo-like kinase Plk2 is an epigenetic determinant of chemosensitivity and clinical outcomes in ovarian cancer. Cancer Res 71: 3317–3327. 21402713 10.1158/0008-5472.CAN-10-2048

[9] Pellegrino R, Calvisi DF, Ladu S, Ehemann V, Staniscia T, et al (2010) Oncogenic and tumor suppressive roles of polo-like kinases in human hepatocellular carcinoma. Hepatology 51: 857–868. 20112253 10.1002/hep.23467

[10] Ward A, Morettin A, Shum D, Hudson JW (2011) Aberrant methylation of Polo-like kinase CpG islands in Plk4 heterozygous mice. BMC Cancer 11: 71. 21324136 10.1186/1471-2407-11-71 PMC3047422

[11] Coley HM, Hatzimichael E, Blagden S, McNeish I, Thompson A, et al (2012) Polo Like Kinase 2 Tumour Suppressor and cancer biomarker: new perspectives on drug sensitivity/resistance in ovarian cancer. Oncotarget 3: 78–83. 22289679 10.18632/oncotarget.332 PMC3292894

[12] de Carcer G, Escobar B, Higuero AM, Garcia L, Anson A, et al (2011) Plk5, a polo box domain-only protein with specific roles in neuron differentiation and glioblastoma suppression. Mol Cell Biol 31: 1225–1239. 21245385 10.1128/MCB.00607-10 PMC3067912

[13] Xie S, Xie B, Lee MY, Dai W (2005) Regulation of cell cycle checkpoints by polo-like kinases. Oncogene 24: 277–286. 15640843 10.1038/sj.onc.1208218

[14] Andrysik Z, Bernstein WZ, Deng L, Myer DL, Li YQ, et al (2010) The novel mouse Polo-like kinase 5 responds to DNA damage and localizes in the nucleolus. Nucleic Acids Res 38: 2931–2943. 20100802 10.1093/nar/gkq011 PMC2875007

[15] Hu M, Polyak K (2008) Microenvironmental regulation of cancer development. Curr Opin Genet Dev 18: 27–34. 18282701 10.1016/j.gde.2007.12.006 PMC2467152

[16] Bapat SA, Jin V, Berry N, Balch C, Sharma N, et al (2010) Multivalent epigenetic marks confer microenvironment-responsive epigenetic plasticity to ovarian cancer cells. Epigenetics 5: 716–729. 20676026 10.4161/epi.5.8.13014 PMC3052886

[17] Campos AC, Molognoni F, Melo FH, Galdieri LC, Carneiro CR, et al (2007) Oxidative stress modulates DNA methylation during melanocyte anchorage blockade associated with malignant transformation. Neoplasia 9: 1111–1121. 18084618 10.1593/neo.07712 PMC2134907

[18] Lim SO, Gu JM, Kim MS, Kim HS, Park YN, et al (2008) Epigenetic changes induced by reactive oxygen species in hepatocellular carcinoma: methylation of the E-cadherin promoter. Gastroenterology 135: 2128–2140 2140 e2121–2128. 18801366 10.1053/j.gastro.2008.07.027

[19] Lu Y, Chu A, Turker MS, Glazer PM (2011) Hypoxia-induced epigenetic regulation and silencing of the BRCA1 promoter. Mol Cell Biol 31: 3339–3350. 21670155 10.1128/MCB.01121-10 PMC3147797

[20] Zochbauer-Muller S, Fong KM, Virmani AK, Geradts J, Gazdar AF, et al (2001) Aberrant promoter methylation of multiple genes in non-small cell lung cancers. Cancer Res 61: 249–255. 11196170

[21] Rofstad EK, Rasmussen H, Galappathi K, Mathiesen B, Nilsen K, et al (2002) Hypoxia promotes lymph node metastasis in human melanoma xenografts by up-regulating the urokinase-type plasminogen activator receptor. Cancer Res 62: 1847–1853. 11912164

[22] Postovit LM, Seftor EA, Seftor RE, Hendrix MJ (2006) Influence of the microenvironment on melanoma cell fate determination and phenotype. Cancer Res 66: 7833–7836. 16912153 10.1158/0008-5472.CAN-06-0731

[23] Shemirani B, Crowe DL (2002) Hypoxic induction of HIF-1alpha and VEGF expression in head and neck squamous cell carcinoma lines is mediated by stress activated protein kinases. Oral Oncol 38: 251–257. 11978547 10.1016/s1368-8375(01)00052-5

[24] Keith B, Johnson RS, Simon MC (2011) HIF1alpha and HIF2alpha: sibling rivalry in hypoxic tumour growth and progression. Nat Rev Cancer 12: 9–22. 22169972 10.1038/nrc3183 PMC3401912

[25] Brigati C, Banelli B, di Vinci A, Casciano I, Allemanni G, et al (2010) Inflammation, HIF-1, and the epigenetics that follows. Mediators Inflamm 2010: 263914. 21197398 10.1155/2010/263914 PMC3010677

[26] McKenzie L, King S, Marcar L, Nicol S, Dias SS, et al (2010) p53-dependent repression of polo-like kinase-1 (PLK1). Cell Cycle 9: 4200–4212. 20962589 10.4161/cc.9.20.13532 PMC3055203

[27] Sosa V, Moline T, Somoza R, Paciucci R, Kondoh H, et al (2013) Oxidative stress and cancer: an overview. Ageing Res Rev 12: 376–390. 23123177 10.1016/j.arr.2012.10.004

[28] Kang KA, Zhang R, Kim GY, Bae SC, Hyun JW (2012) Epigenetic changes induced by oxidative stress in colorectal cancer cells: methylation of tumor suppressor RUNX3. Tumour Biol 33: 403–412. 22274925 10.1007/s13277-012-0322-6

[29] O'Hagan HM, Wang W, Sen S, Destefano Shields C, Lee SS, et al (2011) Oxidative damage targets complexes containing DNA methyltransferases, SIRT1, and polycomb members to promoter CpG Islands. Cancer Cell 20: 606–619. 22094255 10.1016/j.ccr.2011.09.012 PMC3220885

[30] Grewal P, Viswanathen VA (2012) Liver cancer and alcohol. Clin Liver Dis 16: 839–850. 23101985 10.1016/j.cld.2012.08.011

[31] Xie S, Wang Q, Wu H, Cogswell J, Lu L, et al (2001) Reactive oxygen species-induced phosphorylation of p53 on serine 20 is mediated in part by polo-like kinase-3. J Biol Chem 276: 36194–36199. 11447225 10.1074/jbc.M104157200

[32] Morettin A, Ward A, Nantais J, Hudson JW (2009) Gene expression patterns in heterozygous Plk4 murine embryonic fibroblasts. BMC Genomics 10: 319. 19607708 10.1186/1471-2164-10-319 PMC2727538

[33] Ando K, Ozaki T, Yamamoto H, Furuya K, Hosoda M, et al (2004) Polo-like kinase 1 (Plk1) inhibits p53 function by physical interaction and phosphorylation. J Biol Chem 279: 25549–25561. 15024021 10.1074/jbc.M314182200

[34] Martin BT, Strebhardt K (2006) Polo-like kinase 1: target and regulator of transcriptional control. Cell Cycle 5: 2881–2885. 17172872 10.4161/cc.5.24.3538

[35] Swallow CJ, Ko MA, Siddiqui NU, Hudson JW, Dennis JW (2005) Sak/Plk4 and mitotic fidelity. Oncogene 24: 306–312. 15640847 10.1038/sj.onc.1208275

[36] Li J, Tan M, Li L, Pamarthy D, Lawrence TS, et al (2005) SAK, a new polo-like kinase, is transcriptionally repressed by p53 and induces apoptosis upon RNAi silencing. Neoplasia 7: 312–323. 15967108 10.1593/neo.04325 PMC1501148

[37] Nakamura T, Saito H, Takekawa M (2013) SAPK pathways and p53 cooperatively regulate PLK4 activity and centrosome integrity under stress. Nat Commun 4: 1775. 23653187 10.1038/ncomms2752

[38] Zhang H, He J, Li J, Tian D, Gu L, et al (2013) Methylation of RASSF1A gene promoter is regulated by p53 and DAXX. Faseb J 27: 232–242. 23038753 10.1096/fj.12-215491 PMC3528318

[39] Le Gac G, Esteve PO, Ferec C, Pradhan S (2006) DNA damage-induced down-regulation of human Cdc25C and Cdc2 is mediated by cooperation between p53 and maintenance DNA (cytosine-5) methyltransferase 1. J Biol Chem 281: 24161–24170. 16807237 10.1074/jbc.M603724200

[40] Hervouet E, Vallette FM, Cartron PF (2009) Dnmt3/transcription factor interactions as crucial players in targeted DNA methylation. Epigenetics 4: 487–499. 19786833 10.4161/epi.4.7.9883

[41] Berglind H, Pawitan Y, Kato S, Ishioka C, Soussi T (2008) Analysis of p53 mutation status in human cancer cell lines: a paradigm for cell line cross-contamination. Cancer Biol Ther 7: 699–708. 18277095 10.4161/cbt.7.5.5712

[42] van Malenstein H, Gevaert O, Libbrecht L, Daemen A, Allemeersch J, et al (2010) A seven-gene set associated with chronic hypoxia of prognostic importance in hepatocellular carcinoma. Clin Cancer Res 16: 4278–4288. 20592013 10.1158/1078-0432.CCR-09-3274

[43] Burns TF, Fei P, Scata KA, Dicker DT, El-Deiry WS (2003) Silencing of the novel p53 target gene Snk/Plk2 leads to mitotic catastrophe in paclitaxel (taxol)-exposed cells. Mol Cell Biol 23: 5556–5571. 12897130 10.1128/MCB.23.16.5556-5571.2003 PMC166320

[44] Valenti F, Fausti F, Biagioni F, Shay T, Fontemaggi G, et al (2011) Mutant p53 oncogenic functions are sustained by Plk2 kinase through an autoregulatory feedback loop. Cell Cycle 10: 4330–4340. 22134238 10.4161/cc.10.24.18682

[45] Watanabe T, Suzuki T, Natsume M, Nakajima M, Narumi K, et al (2012) Discrimination of genotoxic and non-genotoxic hepatocarcinogens by statistical analysis based on gene expression profiling in the mouse liver as determined by quantitative real-time PCR. Mutat Res 747: 164–175. 22634710 10.1016/j.mrgentox.2012.04.011

[46] Habedanck R, Stierhof YD, Wilkinson CJ, Nigg EA (2005) The Polo kinase Plk4 functions in centriole duplication. Nat Cell Biol 7: 1140–1146. 16244668 10.1038/ncb1320

[47] Vogelstein B, Fearon ER, Hamilton SR, Kern SE, Preisinger AC, et al (1988) Genetic alterations during colorectal-tumor development. N Engl J Med 319: 525–532. 2841597 10.1056/NEJM198809013190901

[48] Matthew EM, Hart LS, Astrinidis A, Navaraj A, Dolloff NG, et al (2009) The p53 target Plk2 interacts with TSC proteins impacting mTOR signaling, tumor growth and chemosensitivity under hypoxic conditions. Cell Cycle 8: 4168–4175. 20054236 10.4161/cc.8.24.10800 PMC2975271

[49] Shahrzad S, Bertrand K, Minhas K, Coomber BL (2007) Induction of DNA hypomethylation by tumor hypoxia. Epigenetics 2: 119–125. 17965619 10.4161/epi.2.2.4613

[50] Ziech D, Franco R, Pappa A, Panayiotidis MI (2011) Reactive oxygen species (ROS)–induced genetic and epigenetic alterations in human carcinogenesis. Mutat Res 711: 167–173. 21419141 10.1016/j.mrfmmm.2011.02.015

[51] Turek-Plewa J, Jagodzinski PP (2005) The role of mammalian DNA methyltransferases in the regulation of gene expression. Cell Mol Biol Lett 10: 631–647. 16341272

[52] Skowronski K, Dubey S, Rodenhiser D, Coomber B (2010) Ischemia dysregulates DNA methyltransferases and p16INK4a methylation in human colorectal cancer cells. Epigenetics 5: 547–556. 20543577 10.4161/epi.5.6.12400 PMC3322492

[53] Park IY, Sohn BH, Choo JH, Joe CO, Seong JK, et al (2005) Deregulation of DNA methyltransferases and loss of parental methylation at the insulin-like growth factor II (Igf2)/H19 loci in p53 knockout mice prior to tumor development. J Cell Biochem 94: 585–596. 15543560 10.1002/jcb.20263

[54] Winkles JA, Alberts GF (2005) Differential regulation of polo-like kinase 1, 2, 3, and 4 gene expression in mammalian cells and tissues. Oncogene 24: 260–266. 15640841 10.1038/sj.onc.1208219

[55] de Carcer G, Manning G, Malumbres M (2011) From Plk1 to Plk5: functional evolution of polo-like kinases. Cell Cycle 10: 2255–2262. 21654194 10.4161/cc.10.14.16494 PMC3230524

[56] Murugan RN, Park JE, Kim EH, Shin SY, Cheong C, et al (2011) Plk1-targeted small molecule inhibitors: molecular basis for their potency and specificity. Mol Cells 32: 209–220. 21809214 10.1007/s10059-011-0126-3 PMC3887635

[57] McInnes C, Wyatt MD (2011) PLK1 as an oncology target: current status and future potential. Drug Discov Today 16: 619–625. 21601650 10.1016/j.drudis.2011.05.002

[58] Garuti L, Roberti M, Bottegoni G (2012) Polo-like kinases inhibitors. Curr Med Chem 19: 3937–3948. 22709006 10.2174/092986712802002455

[59] Joeckel TE, Lubbert M (2012) Clinical results with the DNA hypomethylating agent 5-aza-2′-deoxycytidine (decitabine) in patients with myelodysplastic syndromes: an update. Semin Hematol 49: 330–341. 23079063 10.1053/j.seminhematol.2012.08.001

[60] Herman JG, Graff JR, Myohanen S, Nelkin BD, Baylin SB (1996) Methylation-specific PCR: a novel PCR assay for methylation status of CpG islands. Proc Natl Acad Sci U S A 93: 9821–9826. 8790415 10.1073/pnas.93.18.9821 PMC38513

[61] Li LC, Dahiya R (2002) MethPrimer: designing primers for methylation PCRs. Bioinformatics 18: 1427–1431. 12424112 10.1093/bioinformatics/18.11.1427

[62] Huth A, Vennemann B, Fracasso T, Lutz-Bonengel S, Vennemann M (2013) Apparent versus true gene expression changes of three hypoxia-related genes in autopsy derived tissue and the importance of normalisation. Int J Legal Med 127: 335–344. 23108458 10.1007/s00414-012-0787-2

